# Carbon fibers as active substrates for engineering Ni/Au interfaces toward enhanced hydrogen evolution in ammonia-containing alkaline electrolytes

**DOI:** 10.1038/s41598-026-70216-y

**Published:** 2026-09-05

**Authors:** Annu Pandey, Anton Bjurström, Dhirendra Sahoo, Yuanyuan Li, Valerio Pugliese, Jyoti Shakya, Kåre Tjus, Anna Karlsson, Sudip Chakraborty, Jörgen Ejlertsson, Anders Björk, Richard T. Olsson

**Affiliations:** 1 KTH – Royal Institute of Technology, Department of Fibre and Polymer Technology, School of Chemical Science and Engineering, Teknikringen 56-58, Stockholm, 100 44 Sweden; 2 Wallenberg Initiative Materials Science for Sustainability, Department of Fibre and Polymer Technology, School of Chemical Science and Engineering, KTH Royal Institute of Technology, Teknikringen 56-58, Stockholm, 100 44 Sweden; 3 IVL Swedish Environmental Research Institute, Stockholm, Sweden; 4 Department of Computer Engineering, Modeling, Electronics and Systems (D.I.M.E.S.), University of Calabria, Via P. Bucci, Cubo-42A, Rende, 87036 CS Italy; 5 Nanotechnology Institute (CNR-Nanotec), Via.P. Bucci, Cube 31/c, Rende, 87036 CS Italy; 6 St1 Biokraft, Kungsbron 1, Stockholm, 111 22 Sweden; 7 NKT HV Cables, Technology Consulting, Västerås, SE-72178 Sweden; 8 School of Interdisciplinary Studies & Research, D Y Patil International University, Akurdi, Pune, 411044 India

**Keywords:** Carbon fibers, Electrodeposition, Interfaces, Charge-transfer, Hydrogen evolution reaction, Bimetallic catalysts, Chemistry, Engineering, Materials science

## Abstract

The development of material-efficient electrode platforms for controlling metal dispersion and interfacial charge transfer is central to advanced electrochemical technologies. Here, carbon fibers are used as active substrates for engineering metal–carbon interfaces via thermal activation and sequential electrodeposition. Surface modification exposes graphitic domains and increases roughness, enabling uniform metal nucleation and strong interfacial bonding of the deposited nickel–gold layers, resulting in a hierarchical and highly accessible surface architecture. Electrochemical analysis reveals enhanced charge-transfer behavior and increased electrochemical accessibility, highlighting the role of the carbon scaffold in facilitating electron transport and interfacial charge transfer. The modified fibers exhibit reduced overpotential (168.2 mV at − 10 mA cm⁻²), a Tafel slope of 57 mV dec⁻¹, and decreased charge-transfer resistance (~ 651 Ω), indicating improved interfacial kinetics and more efficient utilization of deposited metals. In an ammonia-containing alkaline electrolyte, a further increase in current response (~ 14–15%) is observed, consistent with a modification of the interfacial reaction environment. The findings demonstrate that carbon fibers function beyond passive supports, acting as conductive and structurally adaptive frameworks that govern metal dispersion, interfacial bonding, and charge-transfer behavior. This work highlights carbon fibers as scalable, material-efficient substrates for designing high-performance metal–carbon hybrid systems for ammonia-containing alkaline electrochemical systems and future alkaline wastewater technologies.

**Supplementary Information:**

The online version contains supplementary material available at https://doi.org/10.1038/s41598-026-70216-y.

## Introduction

The development of advanced electrochemical materials increasingly relies on the ability to control interfacial structure, metal dispersion, and charge-transfer processes across multiple length scales^1–5^. Carbon-based materials have emerged as versatile platforms in this context due to their high electrical conductivity, chemical stability, and tunable surface chemistry^6^. Among them, carbon fibers (CFs) provide several advantages, including a high aspect ratio, mechanical flexibility, and a graphitic microstructure that enables efficient electron transport and structural integrity^7^. These features make CFs attractive substrates for constructing hybrid metal–carbon systems with enhanced functional properties^3^. In parallel, the growing demand for sustainable energy technologies has driven interest in electrochemical processes such as hydrogen evolution from ammonia-containing systems, where electrode materials play a critical role in determining efficiency and stability^1,4^. Platinum remains the benchmark material due to its exceptional catalytic activity; however, its scarcity and high cost have motivated the development of alternative systems with reduced noble metal content^3,6^. Strategies, including nanostructuring and transition-metal-based materials, have been widely explored, yet challenges remain in balancing activity, stability, and material utilization^5,8,9^. In addition, complex electrolyte environments, such as ammonia-containing media, introduce further challenges related to interfacial processes and charge-transfer behavior^10–12^..

Rather than focusing solely on catalyst composition, recent attention has shifted toward understanding the substrate as an active component governing metal dispersion, interfacial architecture, and charge-transfer behavior. In contrast to conventional bulk electrodes such as nickel foam or metal meshes, carbon fibers provide a lightweight, high-surface-area scaffold that enables efficient utilization of deposited metals through enhanced dispersion and interfacial contact^3,6^. Surface treatments such as thermal activation expose graphitic domains, increase defect density, and improve wettability, thereby facilitating controlled nucleation and strong adhesion of metallic coatings. These substrate-induced effects influence the morphology, crystallinity, and electronic properties of the metal–carbon interfaces. Bimetallic systems offer additional opportunities to tailor interfacial behavior through electronic coupling, lattice strain, and synergistic interactions between metallic phases^13,14^. However, achieving uniform deposition and stable integration of multiple metals on fibrous carbon substrates remains challenging, particularly when minimizing noble-metal content while maintaining performance. Nickel is widely used as a transition-metal layer due to its effective interfacial contact with carbon and its ability to form uniform, adherent coatings via electrodeposition^15^. When combined with a thin noble-metal overlayer, such architectures can modify interfacial electronic structure while limiting overall material consumption^16,17^. Despite these advances, the role of carbon fiber substrates in actively controlling metal dispersion, interfacial bonding, and charge-transfer behavior in ammonia-containing alkaline environments remains insufficiently understood.

The present study focuses on establishing the fundamental electrochemical behavior of the developed Ni/Au-coated carbon fiber electrodes in a well-defined ammonia-containing alkaline model electrolyte (1 M KOH + 0.5 M NH₃). The use of a model electrolyte enables systematic investigation of catalyst activity and interfacial charge-transfer processes without the complexity introduced by real industrial wastewater. Evaluation under real alkaline wastewater conditions has subsequently been undertaken in a separate scale-up study and is therefore beyond the scope of the present work. Carbon fibers were used as active substrates for the sequential electrodeposition of layered metal architectures. A transition-metal layer was first deposited on the carbon fiber surface, followed by a thin noble-metal overlayer, creating a well-defined metal-metal interface rather than a bulk alloy. This layered configuration allows controlled modification of interfacial properties while minimizing the use of noble metals. The ability of transition metals to form uniform, adherent coatings on carbon substrates is key to establishing a stable, conductive interface, particularly on complex fibrous morphologies^14,15^. The intrinsic properties of carbon fibers, including high electrical conductivity (~ 10⁴ S m⁻¹), moderate surface area (~ 0.4 m² g⁻¹), and mechanical robustness, facilitated homogeneous metal deposition and efficient electron transport across the interface. Their micrometer-scale diameter and aligned architecture further promoted uniform coating and structural integrity, supporting scalable material design. The structural characterization showed modest changes in the graphitic structure of the carbon fibers following thermal treatment, while electrochemical analysis revealed enhanced charge-transfer behavior and increased electrochemical accessibility. The results demonstrate that the carbon fiber substrate plays an important role in governing metal dispersion while promoting interfacial bonding and improved charge-transfer behavior.

## Experimental

### Chemicals and materials

All chemicals and materials were of high purity and used as received. Nickel sulfate hexahydrate (NiSO₄·6 H₂O, ≥ 98%), sodium sulfate (Na₂SO₄, ≥ 99%), sulfuric acid (H₂SO₄, ≥ 98%), ammonia solution (65%), sodium hydroxide (NaOH, ≥ 95%), and potassium hydroxide (KOH, 1 M) were obtained from Sigma-Aldrich. Boric acid (H₃BO₃, ≥ 99%) was obtained from Alfa Aesar. The electrode carbon fiber materials used were PYROFIL™ TOW (TR 30 S 3 L, A2), sourced from Japan and derived from the carbonization of the polyacrylonitrile (PAN) polymer. The platinum wire counter electrode, gold chloride trihydrate (HAuCl₄·3 H₂O), and potassium chloride (KCl) were obtained from Sigma-Aldrich (Sweden).

### Preparation of carbon fibers (CF)

Approximately 1 m of carbon fiber was desized (stripped from its protective epoxy coating) using a shock-heating method. The fiber was connected across a variable autotransformer (METREL HSN0203, 1.82 kVA), with each end linked to the positive and negative terminals, respectively. A rapid voltage ramp increased the fiber temperature to approximately 1200 °C for 3 s, effectively thermally degrading the polymer coating^3^. Complete removal was confirmed by microscopy after heat exposure, which produced a uniform orange glow with visible smoke (consistent with a temperature > 1000 °C), indicating the formation of a clean, polymer-free carbon fiber surface.

### Preparation of Nickel-coated carbon fibers (Ni-CFs)

**Fig. 1:**
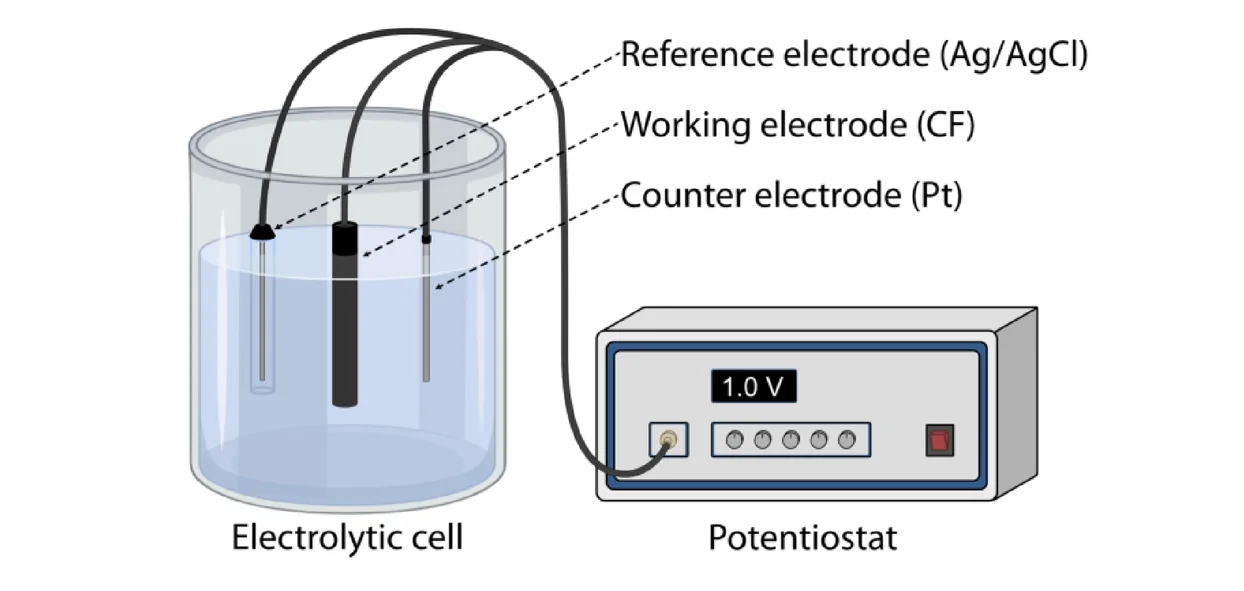
Schematic illustration of the electrodeposition setup, showing a single-compartment electrochemical cell with carbon fibers as the working electrode, a platinum wire as the counter electrode, and an Ag/AgCl reference electrode.

A cylindrical electrolytic cell (105 × 50 × 60 mm) was used for the nickel electrodeposition experiments. A 100 mL electrolyte was prepared by dissolving 0.05 M NiSO₄·6 H₂O and 14 g L⁻¹ H₃BO₃ in deionized Milli-Q water. Boric acid was used as a buffering agent to stabilize the electrolyte during electrodeposition and promote the formation of a uniform and adherent nickel coating. The electrolyte pH was adjusted to 3.5 using dilute H₂SO₄ or NaOH before deposition. The deposition bath temperature was maintained at 40 °C throughout the experiment. As illustrated in Fig. 1, the electrodeposition system consisted of a single-compartment electrolytic cell connected to a DC power supply. The thermally desized carbon fiber served as the cathode, while a platinum wire was used as the anode. Nickel electrodeposition was carried out under potentiostatic conditions at a constant applied potential of 4.0 V for 10 min. These deposition parameters were selected based on systematic optimization of deposition potential, electrolyte pH, temperature, and deposition time, resulting in a uniform, adherent, and highly conductive nickel coating suitable for subsequent gold electrodeposition.

### Preparation of gold-coated carbon fibers (Ni/Au/CF)

The electrodeposition of gold onto the nickel-coated carbon fibers was performed using a potentiostatic method to achieve a uniform and well-adhering gold layer. The precursor solution was prepared by dissolving HAuCl₄·3 H₂O in 0.1 M KCl. The nickel-coated carbon fibers were then immersed in this electrolyte solution, serving as the working electrode for the electrodeposition process. A clean platinum wire and an Ag/AgCl electrode were employed as the counter and reference electrodes, respectively. The electrodeposition was carried out for 3600 s at −0.3 V with a 3 mM HAuCl₄ concentration. The Ni/Au-coated carbon fibers were thoroughly rinsed with deionized water to remove any residual electrolyte or unreacted precursors. The obtained Ni/Au/CF samples were then subjected to structural and electrochemical characterization using microscopy and spectroscopic techniques, as well as electrochemical analysis.

### Structural and electrochemical characterization

#### Scanning Electron Microscopy (SEM)

To investigate morphological and chemical changes during the surface treatment and metal deposition process, untreated, treated, and metal-coated carbon fibers were characterized using a Hitachi SEM S-4800 (Japan), equipped with an energy-dispersive X-ray spectroscopy (EDS) detector (X-Max 80 SDD, Oxford Instruments, UK), to examine surface morphology, coating uniformity, and elemental composition of the deposited metals.

#### Raman spectroscopy

Raman spectra were collected using a Horiba Jobin Yvon LabRAM HR800 Evolution confocal Raman spectrometer, equipped with a 532 nm excitation laser and an Olympus MPlan N 100x objective, focused to a ~ 1 μm spot. A 600 g mm^-¹^ grating was used to achieve a spectral resolution of approximately 2 cm^-^¹, allowing precise identification of graphitic and disordered carbon features.

#### X-ray diffraction (XRD)

XRD analysis was carried out on a PANalytical X’Pert Pro diffractometer using a Cu Kα radiation source to determine the crystalline phases and confirm the presence of deposited metallic nickel (Ni) and gold (Au).

#### Electrochemical characterization

The electrocatalytic performance of the metal-coated thermally desized carbon fiber electrodes was evaluated using a Metrohm Autolab PGSTAT204 potentiostat/galvanostat (Metrohm Autolab B.V., The Netherlands) in a conventional three-electrode configuration. The prepared Ni/Au-carbon fiber served as the working electrode, a platinum wire as the counter electrode, and a saturated calomel electrode (SCE) as the reference electrode. All electrochemical measurements were normalized using the exposed geometric area of the immersed carbon fiber electrode (1 cm²), and the results are reported as current density (mA cm⁻²). Because the exposed geometric area was 1 cm², the measured current values were numerically identical to the reported current densities (mA cm⁻²). Overpotentials were determined at a current density of − 10 mA cm⁻², and all electrodes were evaluated under identical experimental conditions. Electrochemical performance was evaluated in a well-defined ammonia-containing alkaline model electrolyte consisting of 1 M KOH and 0.5 M NH₃. This electrolyte was selected to investigate the intrinsic catalytic behavior and interfacial charge-transfer characteristics under controlled laboratory conditions while avoiding the compositional variability associated with real industrial wastewater.

Electrochemical measurements were performed in 1 M KOH containing 0.5 M NH₃ to evaluate the hydrogen evolution reaction (HER) performance. Cyclic voltammetry (CV), linear sweep voltammetry (LSV), and electrochemical impedance spectroscopy (EIS) were carried out to investigate the electrochemical behavior, catalytic activity, charge-transfer characteristics, and HER kinetics of the prepared electrodes. EIS measurements were conducted over a frequency range of 10 mHz to 100 kHz. A solution containing 1 mM K₃[Fe(CN)₆] and 0.1 M KCl was used as a standard reversible redox probe to evaluate the influence of thermal desizing and metal deposition on the electron-transfer characteristics of the carbon fiber electrodes. This electrolyte was selected for electrode characterization because it provides a well-defined outer-sphere redox system with reproducible electrochemical behavior. The electrocatalytic HER performance was subsequently evaluated in 1 M KOH containing 0.5 M NH₃, which represents the desired operating electrolyte.

All polarization curves were corrected for the uncompensated solution resistance (iR compensation) before analysis. The measured potentials were converted to the reversible hydrogen electrode (RHE) scale using:

E_RHE_=E_SCE_ + 0.241 + 0.0591 × pH.

The HER overpotential (η) was determined from the iR-corrected polarization curves as the potential required to achieve a current density of − 10 mA cm⁻². Unless otherwise stated, all electrochemical measurements were independently performed in triplicate (*n* = 3), and the reported values represent the mean of three independent experiments. The reference electrode was positioned close to the working electrode to minimize the uncompensated solution resistance.

**Fig. 2:**
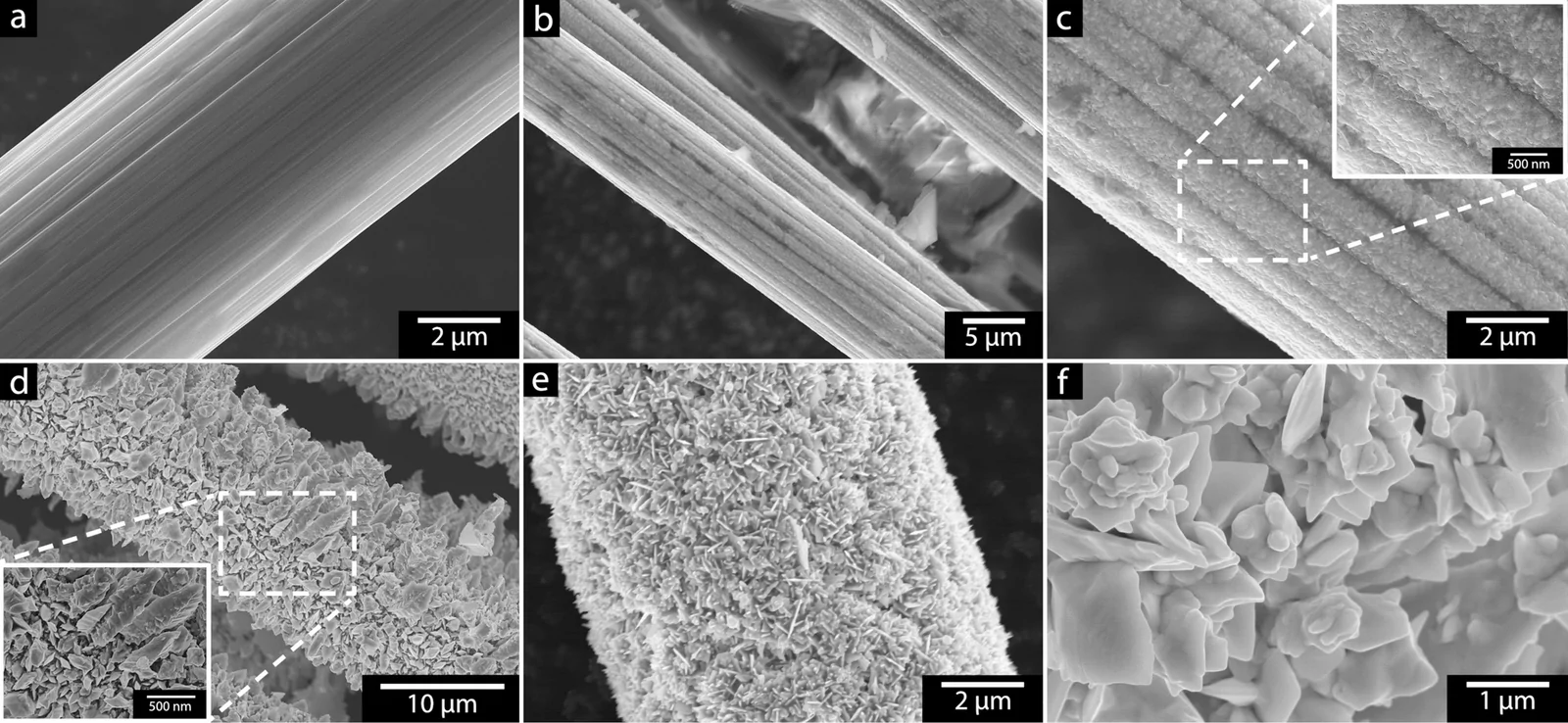
Scanning electron microscopy (SEM) images of (a) heat-treated carbon fibers (CF), (b-c) nickel-coated carbon fibers (Ni/CF), and (d-f) nickel-gold-coated carbon fibers (Ni/Au/CF). The images show changes in surface morphology at different magnifications, including longitudinal grooves on CF and nanoscale features on metal-coated fibers.

## Results and discussion

### Electrochemical deposition of catalytically active metallic coatings on carbon fibers

The surface morphology of carbon fibers at different processing stages, including thermal desizing, primary nickel (Ni) metal deposition, and subsequent gold (Au) overlayer formation, was investigated using scanning electron microscopy (SEM), as shown in Fig. 2. Following the thermal treatment, the carbon fibers exhibited a smooth surface (Fig. 2a), confirming the effective removal of the polymer sizing layer (coating). The exposed surface retained its longitudinal grooves associated with the filament drawing process of polyacrylonitrile (PAN)-derived fibers, originally carbonized at temperatures exceeding 1800 °C ^17^. The surface roughness post-thermal treatment enhanced the exposure of graphitic domains and provided energetically favorable nucleation sites for metal deposition, improving interfacial adhesion.

Deposition of the first metallic layer yielded a very thin conformal metal coating that closely followed the fiber morphology (Figs. 2b-c). At higher magnification, the surface consisted of uniformly distributed nanoscale grain features with an average size of approximately 40 nm, indicative of controlled nucleation and growth. This morphology is characteristic of diffusion-limited electrodeposition and leads to a significant increase in electrochemical accessibility^18^. Importantly, the fibrous structure of the carbon substrate remained intact, demonstrating that the coating process retained the structural integrity of the carbon fibers while enabling uniform metal coverage. Subsequent deposition of the secondary metal layer produced a hierarchical nanostructured surface (Figs. 2d-f), composed of finer nanoscale features and larger flake-like, or prism-shaped, crystallites with lateral dimensions up to ~ 500 nm. Compared to direct deposition of gold onto bare carbon fibers, the presence of the intermediate nickel layer enabled more uniform nucleation and reduced particle aggregation, indicating substrate-mediated control of the metal coating growth. This behavior is consistent with island-type nucleation mechanisms commonly observed during noble metal deposition on heterogeneous substrates^19,20^.

**Fig. 3:**
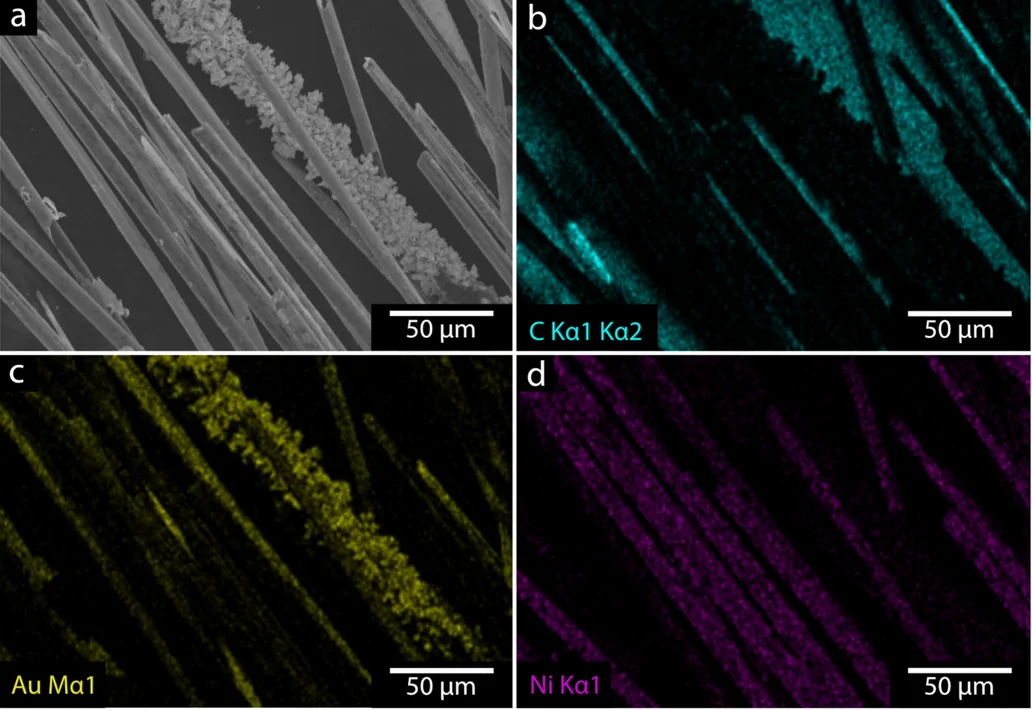
SEM image of carbon fibers with corresponding energy-dispersive X-ray spectroscopy (EDX) elemental mapping showing the spatial distribution of nickel (Ni) and gold (Au) on the fiber surface. The strong signals for Ni (23.5 wt%) and Au (16.9 wt%) in the EDX spectrum validated the formation of a dual-layer coating.

To confirm and evaluate the extent and distribution of nickel and gold on the carbon fiber (CF) surfaces, Energy-Dispersive X-ray Spectroscopy (EDX) was performed. EDX analysis confirmed successful nickel deposition, with Ni accounting for 23.5 wt% of the detected elemental composition (Fig. 3). This high concentration confirmed successful deposition of the nickel layer onto the carbon fibers, serving as an adhesion-promoting intermediate layer in the surface engineering of the CF. The relatively uniform Ni elemental distribution observed by EDX was consistent with the uniform coating morphology observed by SEM, suggesting that the deposition conditions effectively achieved high surface coverage across the carbon fiber surfaces. The use of nickel as an intermediate layer is particularly important when introducing noble metals like gold, as it improves adhesion and mitigates selective deposition in areas that typically promote direct diffusion onto the already-formed metallic deposits and the formation of local particles and aggregates on the carbon surface. Gold was also evident in the EDX spectrum, accounting for 16.9 wt% of the total composition. This confirmed that a second gold layer was successfully deposited over the nickel-coated CFs. The relatively high Au content confirmed successful deposition of the secondary gold layer and was consistent with the nanoscale surface morphology observed by SEM (Figs. 2e, f).

The combined EDX detection of both Ni and Au was possible (with no apparent signal suppression), suggesting that the layers were distinct yet thin enough to allow partial X-ray signal contributions from both metals. EDX detection of surface elements, typically within 1–2 μm, depends on the accelerating voltage and material density. Overall, the resulting gold coating not only smoothed out larger nickel surface features but also introduced a porous, hierarchical nanoscale texture (Figs. 2d–f). This nanostructured morphology was expected to increase electrochemically accessible surface sites and facilitate improved charge-transfer kinetics. The overall observations demonstrated that the carbon fiber substrate plays an important role in governing metal nucleation, growth, and spatial distribution. The thermal treatment enhanced the mechanical interlocking and exposed active graphitic sites, whereas the fibrous architecture promoted uniform coating and hierarchical structuring. This substrate-driven control over morphology is critical in achieving high surface accessibility and efficient interfacial charge transfer in these metal-carbon hybrid systems. The Ni/Au interface is expected to promote efficient interfacial charge transfer, whereas Au may also contribute to enhanced corrosion resistance. To further understand the chemical structure and crystalline composition of the deposited layers, Raman spectroscopy and X-ray diffraction (XRD) analyses were carried out and are presented in the following sections. Notably, the absence of large agglomerates or patchy regions in the Au layer reflected high uniformity of the electrodeposition process.

### Raman spectroscopy of structural changes with surface treatment

**Fig. 4:**
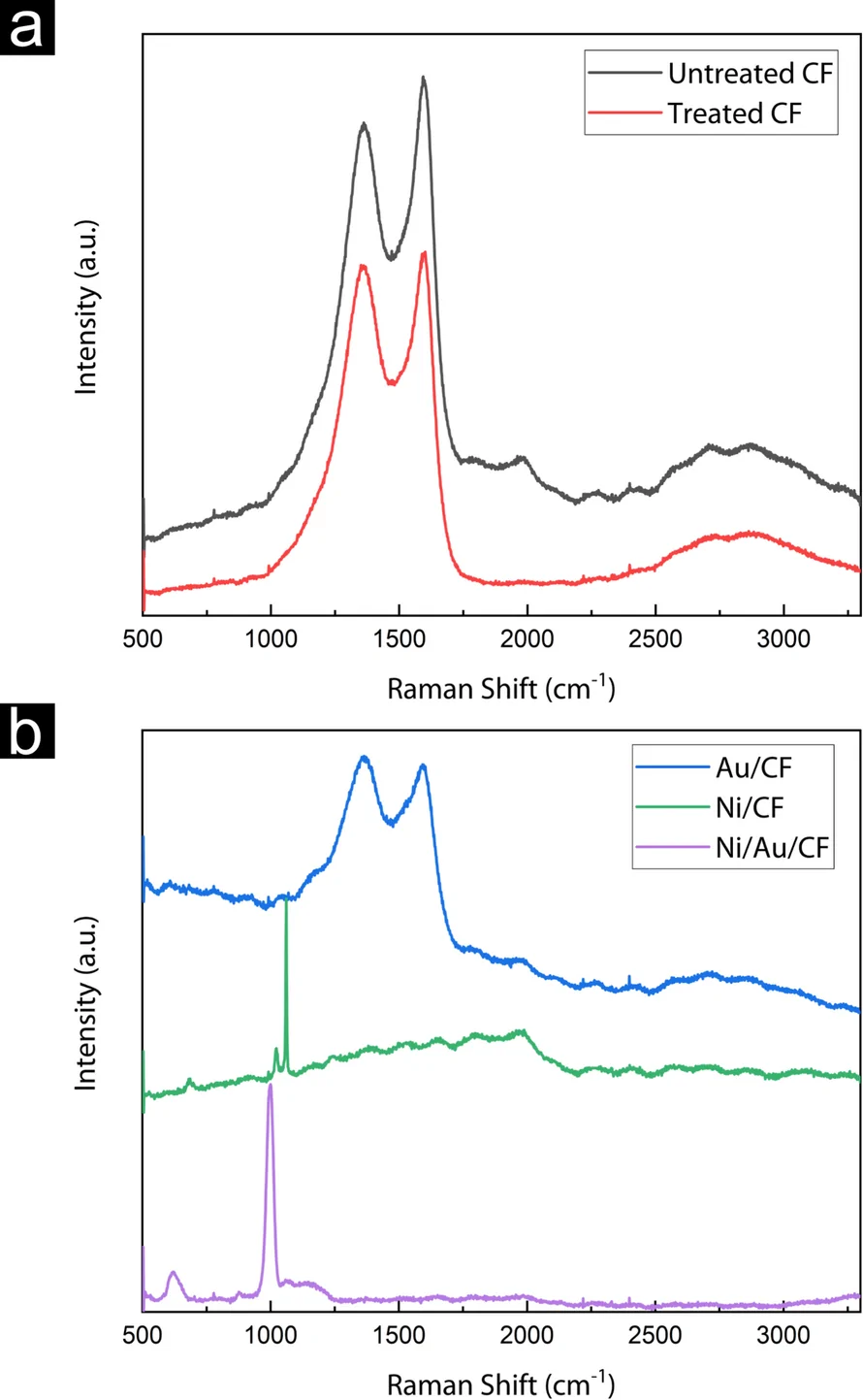
Raman spectra illustrating the structural evolution of the carbon fibers during electrode preparation. (a) Comparison of untreated (UTCF) and thermally desized (HVCF) carbon fibers showing the characteristic D and G bands. (b) Raman spectrum of the final Ni/Au-coated carbon fiber electrode. The baseline-corrected Raman spectra and the corresponding I_D_/I_G_ analysis are provided in Fig. S1 (Supporting Information).

To investigate the structural evolution of the carbon fibers following thermal desizing and subsequent metal electrodeposition, Raman spectroscopy was performed, as shown in Fig. 4. To facilitate direct comparison, the Raman spectra of the untreated carbon fiber (UTCF) and thermally desized carbon fiber (HVCF) are presented in Fig. 4a, whereas the Raman spectrum of the final Ni/Au-coated carbon fiber electrode is shown separately in Fig. 4b. The baseline-corrected Raman spectra together with the corresponding I_D_/I_G_ analysis are provided in the Supporting Information (Fig. S1).

Figure 4 shows the Raman spectra illustrating the structural evolution of the carbon fibers during electrode preparation; comparison of untreated (UTCF) and thermally desized (HVCF) carbon fibers showing the characteristic D and G bands (a), and the Raman spectrum of the final Ni/Au-coated carbon fiber electrode (b). The Raman spectra exhibited the characteristic D band (≈ 1350 cm⁻¹) associated with structural disorder and defects in the graphitic lattice, and the G band (≈ 1580 cm⁻¹) corresponding to the in-plane vibration of sp²-hybridized carbon atoms in graphitic domains. These two bands are widely used to evaluate structural ordering and defect density in carbon-based materials. To provide a more quantitative assessment of the structural changes, the Raman spectra were processed using an anchor-point spline baseline correction to minimize the fluorescence background before evaluating the I_D_/I_G_ ratios. The baseline-corrected Raman spectra and the corresponding numerical I_D_/I_G_ ratios are presented in Fig. S1. The analysis revealed a modest change in the I_D_/I_G_ ratio following thermal desizing compared with the untreated carbon fiber, consistent with partial removal of the surface sizing layer and slight improvement in the structural ordering of the exposed carbon surface. In contrast, no significant difference in the I_D_/I_G_ ratio was observed between the thermally desized and Ni/Au-coated carbon fibers, suggesting that the electrodeposition process does not substantially affect the underlying graphitic structure of the carbon substrate. Therefore, the enhanced electrochemical performance of the Ni/Au-coated carbon fiber could be attributed to the formation of catalytically active Ni/Au interfaces, extensive metal dispersion, and interfacial charge-transfer characteristics rather than further graphitization of the carbon support. The interpretation is consistent with the electrochemical results, including the reduced charge-transfer resistance, lower HER overpotential, and improved reaction kinetics observed after sequential Ni/Au electrodeposition .

### X-ray diffraction analysis of the CF sample phase structures

**Fig. 5:**
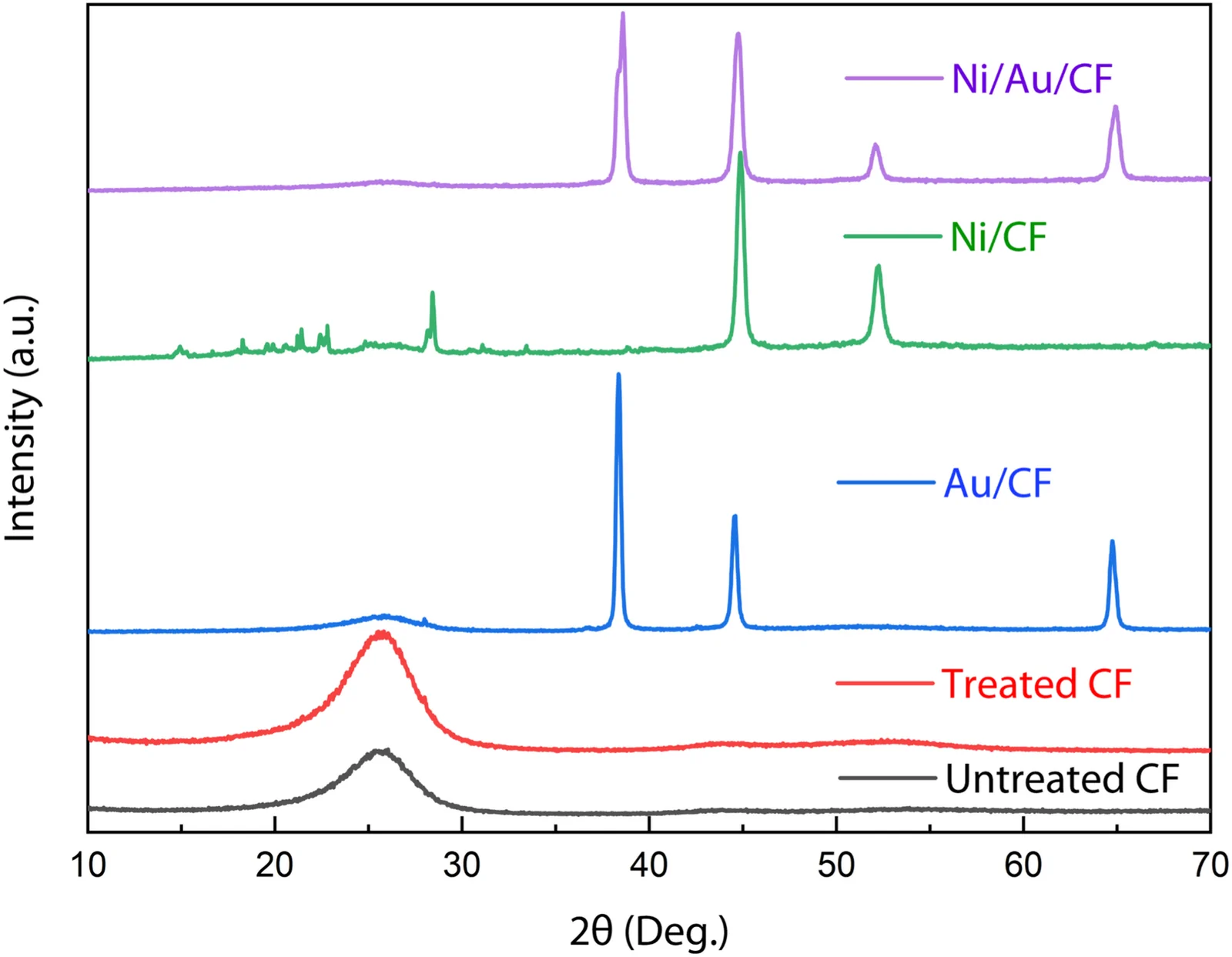
X-ray diffraction (XRD) patterns of untreated CF, thermally treated CF, Ni-coated CF, and Ni/Au/CF, showing diffraction peaks corresponding to graphitic carbon and deposited metallic phases. The narrower, slightly shifted reflections in Ni/Au/CF were consistent with enhanced crystallinity and lattice strain associated with interfacial interactions between the deposited Ni and Au phases.

X-ray diffraction (XRD) analysis was performed as shown in Fig. 5 to assess the crystallinity and phase evolution of the carbon fiber samples at different processing stages: untreated CF (UTCF), thermally treated (HVCF), Ni-coated CF, and Ni/Au-coated CF. The UTCF exhibited a broad, low-intensity diffraction peak centered around 2θ ≈ 25°, corresponding to the (002) plane of disordered graphitic carbon^7^. This broad feature is indicative of an amorphous structure with low graphitic order.

**Fig. 6:**
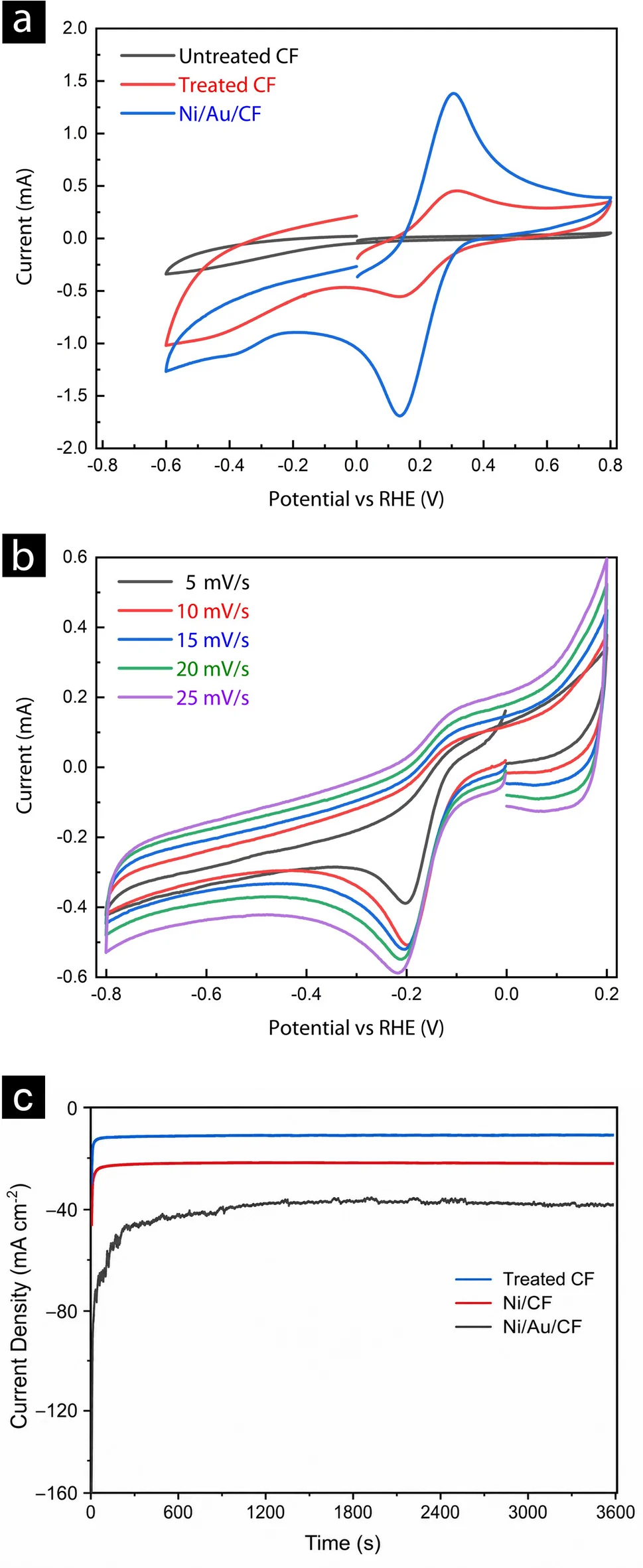
(**a**) Cyclic voltammetry curves of untreated carbon fibre (UTCF), thermally treated carbon fibre, and Ni/Au-coated carbon fibre recorded in 1 mM potassium ferricyanide with 0.1 M KCl at a scan rate of 50 mV s ¹. (**b**) Cyclic voltammograms of Ni/Au-coated carbon fibre recorded at scan rates ranging from 5 to 25 mV s ¹. (**c**) Short-term chronoamperometric responses of thermally treated carbon fibre, Ni/CF, and Ni/Au/CF electrodes recorded at an applied potential of 0.4 V vs. Ag/AgCl in **1** M KOH containing 0.5 M NH₃ for 3600 s.

After the thermal treatment, the CF showed sharpening of the (002) diffraction peak. The sharpening was consistent with a modest increase in the structural ordering of the carbon substrate, potentially associated with the removal of amorphous surface carbon and contaminants. This result was consistent with the Raman analysis, which showed improved graphitic ordering after the thermal treatment. Upon the electrodeposition of the nickel, additional sharp reflections emerged at 2θ ≈ 44.5°, attributed to the (111) planes of face-centered cubic (FCC) Ni^21^. These reflections confirmed the successful formation of a crystalline nickel phase on the carbon fiber surface. In the Ni/Au/CF sample, new peaks appeared at 2θ ≈ 38.2°, corresponding to the (111) planes of FCC gold^20^. The sharpness and intensity of these peaks reflected the high crystallinity of the deposited Au layer. In the Ni/Au/CF sample, both Ni and Au diffraction peaks were narrower and showed slight shifts compared to those in the single-metal-coated samples. Such shifts are consistent with lattice strain or compressive stress at the Ni/Au interface, suggesting that lattice strain arose from strong Ni/Au interfacial interactions rather than definitive alloy formation. The effects may alter the local electronic environment, thereby influencing catalytic activity and stability under electrochemical conditions. Following sequential Ni/Au electrodeposition, the diffraction pattern became dominated by the crystalline metallic phases, while the relative intensity of the graphite (002) reflection decreased. As also confirmed by the SEM images, the carbon fibers were covered by a continuous metallic coating, and the increased contribution from the Ni and Au layers resulted in the metallic reflections dominating the diffraction pattern. Accordingly, the weaker graphite peak was attributed to the coating thickness and sample geometry rather than structural degradation or disappearance of the underlying carbon substrate. The XRD patterns were not normalized, and the observed peak intensities reflect the relative contributions of the carbon substrate and the deposited metallic phases. Overall, the XRD results confirmed increased structural ordering following thermal treatment and the formation of well-defined crystalline Ni and Au phases after sequential electrodeposition. The observed changes in the metallic reflections were consistent with lattice strain and interfacial interactions between the deposited Ni and Au phases. To correlate these structural modifications with electrochemical performance, cyclic voltammetry (CV) and related electrochemical measurements were carried out using a standard redox probe system (Fig. 6). Further, to evaluate changes in surface reactivity, charge-transfer kinetics across the differently treated carbon fiber electrodes were measured.

### Effect of surface modification on the electrochemical activity of the carbon fiber electrodes

The influence of surface modification on the electrochemical response of carbon fiber electrodes was evaluated by cyclic voltammetry in 1 mM potassium ferricyanide containing 0.1 M KCl at a scan rate of 50 mV s ¹, see Fig. 6a. The untreated carbon fibers exhibited a weak and broad voltammetric response with low current density, indicative of sluggish electron-transfer kinetics. This behavior was attributed to the insulating epoxy coating (sizing), which limited electrical contact and restricted access of redox species to the underlying conductive carbon surface. Following the thermal treatment, an increase in both anodic and cathodic currents was observed, along with a more pronounced capacitive background. This enhancement is consistent with the removal of the surface coating and the exposure of graphitic domains, which improved wettability and facilitated electrolyte penetration. The resulting increase in electrochemical accessibility enabled more efficient interaction between the electrode surface and the redox probe. The metal-coated carbon fibers exhibited the highest current response among all samples. Although the voltammograms did not display sharp, diffusion-controlled peaks typical of planar electrodes, a more pronounced redox response with higher current density was observed. This response is consistent with enhanced interfacial charge transfer and a higher density of electrochemically accessible sites arising from the nanostructured metal coating. The oxidation and reduction peaks observed in the cyclic voltammograms correspond to the reversible Fe (CN)₆³⁻/Fe (CN)₆⁴⁻ redox couple. The increase in peak current after thermal desizing and Ni/Au electrodeposition is consistent with improved electron-transfer behavior and enhanced electrochemical accessibility of the modified carbon fiber electrodes. These measurements were used to compare the electrochemical response of the different electrodes following surface modification. In summary, the voltammetric profiles were dominated by capacitive and pseudocapacitive contributions, which are characteristic of high-surface-area fibrous electrodes. This behavior arises from non-ideal diffusion, partial adsorption of ferricyanide species, and the heterogeneous nature of the metal-carbon interface. Despite the non-ideal voltammetric characteristics associated with fibrous porous electrodes, the progressive increase in current response clearly demonstrated the significant role of surface engineering in improving interfacial accessibility and charge-transfer kinetics.

### Stability and kinetic analysis of the Ni/Au carbon fibers

The kinetic response and electrochemical stability of the modified carbon fiber electrodes were evaluated using cyclic voltammetry at scan rates ranging from 5 to 25 mV s ¹, see Fig. 6b. Across this range, the voltammograms exhibited a consistent capacitive-dominated profile, characteristic of high-surface-area fibrous electrodes with nanostructured metallic coatings. As the scan rate increased, both anodic and cathodic currents scaled proportionally with the applied scan rate, indicating that the interfacial processes were well-defined and kinetically accessible. This linear dependence is typical of surface-controlled charge-transfer and capacitive contributions, reflecting efficient electron transport across the metal–carbon interface. Although distinct diffusion-controlled redox peaks were not observed, as expected for porous and heterogeneous electrode systems, the overall shape of the voltammograms remained preserved across all scan rates. The absence of significant peak shifts or distortion suggested that the interfacial structure remained stable, with no evidence of passivation, delamination, or degradation within the investigated potential window. The reproducibility of the voltammetric response across varying scan rates further confirmed the robustness of the metal–carbon interface and indicated that the deposited layers maintained electrical connectivity and structural integrity under dynamic polarization conditions. This behavior highlights the carbon fiber substrate’s ability to support stable, uniform metal coatings while facilitating rapid charge-transfer processes.

Figure 6c shows the chronoamperometric responses of the thermally desized carbon fiber (HVCF), Ni/CF, and Ni/Au/CF electrodes, respectively. All three electrodes showed an initial decrease in cathodic current, followed by a nearly steady current throughout the measurement. Formation of the electrode/electrolyte interface, including EDLC, stabilization of the interfacial reaction environment, and hydrogen bubble nucleation during the onset of electrolysis, was mainly responsible for the initial transient of current density. After this initial stabilization, the Ni/Au/CF electrode maintained the largest steady-state cathodic current density, indicating enhanced electrocatalytic activity under continuous polarization. The stable current response suggested that the sequentially deposited Ni/Au coating remained electrochemically active without appreciable deterioration over the measurement period. The superior performance of the Ni/Au/CF electrode was consistent with its lower overpotential, smaller Tafel slope, and reduced charge-transfer resistance, demonstrating that the engineered Ni/Au/CF facilitated more efficient interfacial charge transfer and reaction kinetics compared to Ni/CF and thermally desized carbon fiber (HVCF) electrodes.

### Interfacial charge-transfer behavior and electrolyte-dependent kinetics

**Fig. 7:**
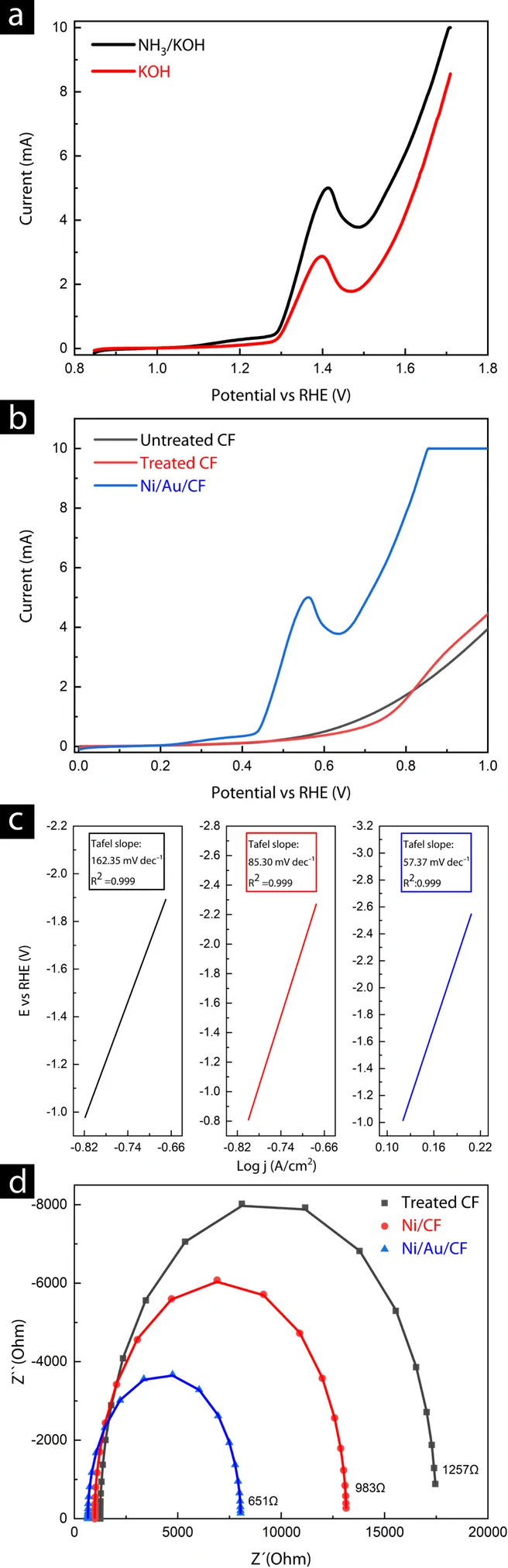
(**a**) Linear sweep voltammograms of the Ni/Au-coated carbon fiber (Ni/Au/CF) recorded in 1 M KOH and 1 M KOH containing 0.5 M NH₃. (**b**) Comparison of the LSV-derived HER polarization curves of untreated carbon fiber (UTCF), thermally desized carbon fiber (HVCF), and Ni/Au-coated carbon fiber (Ni/Au/CF) recorded in 1 M KOH containing 0.5 M NH₃. (**c**) Tafel plots derived from the corresponding polarization curves for untreated, thermally treated, and Ni/Au-coated carbon fibers. (**d**) Nyquist plots from electrochemical impedance spectroscopy (EIS) measurements over a frequency range of 10–100 kHz for untreated, thermally treated, and Ni/Au-coated carbon fibers.

The electrochemical measurements were performed using a model ammonia-containing alkaline electrolyte to establish the intrinsic electrocatalytic behavior of the developed electrodes under controlled conditions. Although real industrial alkaline wastewater contains additional dissolved salts, organic compounds, and trace contaminants, etc., that influence reaction kinetics, the model electrolyte provided a platform for isolating reproducible catalyst-related effects before subsequent validation in real wastewater systems. Figure 7 summarizes the electrocatalytic hydrogen evolution reaction (HER) performance of the carbon fiber electrodes, illustrating both the influence of ammonia on the Ni/Au-coated electrode and the effect of sequential surface modification on the electrochemical response.

Figure 7a shows the HER performance of the Ni/Au-coated carbon fiber (Ni/Au/CF) in 1 M KOH and 1 M KOH containing 0.5 M NH₃, highlighting the influence of ammonia on the electrochemical response of the metal catalyst. The interfacial charge-transfer behavior was evaluated using linear sweep voltammetry (LSV). In 1 M KOH, the current density increased gradually at more negative potentials, reflecting the activation of interfacial charge-transfer processes associated with water reduction. Upon the addition of 0.5 M NH₃, a systematic increase in current density was observed across the entire potential range, accompanied by a slight shift in the onset potential toward less negative values. At − 1.70 V vs. RHE, the current density increased from approximately 9.2 to 10.5 mA cm⁻², corresponding to an enhancement of about 14–15%. Subtle changes in the slope within the intermediate potential region (− 1.30 to − 1.45 V vs. RHE) further indicated modifications in interfacial kinetics rather than the emergence of a new reaction pathway. Under the present conditions, the results suggested that ammonia modified the interfacial electrolyte environment, which may have facilitated water dissociation and improved HER kinetics. The present data do not establish direct catalytic participation of ammonia. Consequently, the enhanced current response is attributed to improved charge-transfer kinetics and more favorable formation and stabilization of adsorbed hydrogen intermediates (H*), rather than a distinct ammonia-assisted reaction mechanism^22^.

Figure 7b compares the HER performance of untreated carbon fiber (UTCF), thermally desized carbon fiber (HVCF), and Ni/Au-coated carbon fiber (Ni/Au/CF) in 1 M KOH containing 0.5 M NH₃, demonstrating the effect of sequential surface modification on electrocatalytic activity. The untreated carbon fiber exhibited the lowest current response because of limited interfacial charge transfer and poor surface accessibility. Thermal treatment significantly enhanced the electrochemical response by removing insulating surface layers and exposing conductive graphitic domains, thereby reducing interfacial resistance. The Ni/Au-coated carbon fiber displayed the highest current density, confirming that metal deposition further improved electrochemical accessibility and electronic coupling at the metal–carbon interface, resulting in superior HER activity.

The overpotential required to reach a benchmark current density of − 10 mA cm⁻² decreases progressively from 198.6 mV UTCF to 179.5 mV HVCF) and further to 168.2 mV for the Ni/Au/CF, confirming that surface activation and controlled metal deposition enhance interfacial charge-transfer efficiency^13,14,18^.

Further insight into the reaction kinetics was obtained from Tafel analysis (Fig. 7c). The modified carbon fibres (Ni/Au-coated carbon fibers) exhibited a Tafel slope of 57.37 mV dec⁻¹, significantly lower than that of treated (85.30 mV dec⁻¹) and untreated fibers (162.35 mV dec⁻¹), indicating faster interfacial kinetics. A slope near ~ 60 mV dec⁻¹ is commonly associated with a Volmer–Heyrovsky-type process, where electrochemical desorption is rate-limiting, suggesting that the engineered interface is consistent with improved interfacial kinetics under alkaline conditions^9,22^.

Electrochemical impedance spectroscopy (EIS) further supported these observations (Fig. 7d). The modified fibers exhibited the smallest semicircle diameter, corresponding to the lowest charge-transfer resistance (Rct ≈ 651 Ω) among Ni/Au-coated carbon fibers, compared to 983 Ω for treated fibers and 1257 Ω for untreated fibers. This reduction in resistance indicated reduced interfacial charge-transfer resistance and more efficient electron transfer across the metal- carbon interface. EIS measurements were performed at − 0.4 V vs. SCE in 1 M KOH containing 0.5 M NH₃. The measured charge-transfer resistance reflects the overall impedance of the fibrous electrode/electrolyte interface, including contributions from the carbon fiber substrate, deposited metallic coating, interfacial contact resistance, electrolyte penetration within the fibrous architecture, and gas-evolution-related interfacial effects. Therefore, the absolute R_ct_ values should be interpreted as characteristics of the complete electrode system rather than the intrinsic conductivity of the metallic coating alone. The progressive decrease in R_ct_ after thermal desizing and Ni/Au electrodeposition indicates improved interfacial electron-transfer kinetics, consistent with the LSV and Tafel results.

The enhanced HER performance of the Ni/Au-coated carbon fiber electrode cannot be attributed solely to an increased surface area. Instead, it results from the synergistic effects of thermal desizing, improved metal dispersion following sequential Ni/Au electrodeposition, and enhanced interfacial charge-transfer kinetics. These effects are evidenced by the reduced charge-transfer resistance, lower overpotential, and smaller Tafel slope, indicating that the improved electrocatalytic activity primarily arises from enhanced charge-transfer efficiency and the intrinsic catalytic properties of the Ni/Au heterostructure.

Overall, these results demonstrate that the electrochemical response is governed by substrate-induced control of interfacial structure and electronic coupling. Thermal activation enhances surface accessibility, while metal deposition introduces a hierarchical architecture that promotes efficient charge transfer. The presence of ammonia further suggests that ammonia may influence the local interfacial environment and intermediate behavior without altering the fundamental reaction pathway. Together, these effects highlight the critical role of carbon fiber substrates in controlling interfacial kinetics and enabling efficient utilization of deposited metals in hybrid electrochemical systems.

## Conclusions

In this work, carbon fibers are demonstrated to serve as active substrates for the controlled formation of metal–carbon interfaces via sequential electrodeposition. Thermal desizing induced modest changes in the structural organization of the carbon substrate, as indicated by Raman analysis, consistent with decreased surface disorder. SEM and XRD analyses confirmed the formation of uniform, crystalline metal layers, resulting in a hierarchical surface architecture with increased roughness and interfacial accessibility. The electrochemical measurements revealed that surface modification enhances interfacial charge-transfer behavior. The modified fibers exhibit reduced overpotential (168.2 mV at − 10 mA cm⁻²), lower charge-transfer resistance (≈ 651 Ω), and faster interfacial kinetics compared to untreated and thermally treated substrates. Tafel analysis (57.37 mV dec⁻¹) is consistent with enhanced adsorption–desorption dynamics characteristic of a Volmer–Heyrovsky-type process. The results further indicate that electrolyte composition influences interfacial behaviour, with the presence of ammonia being associated with a more favourable electrochemical response for HER. Overall, this study demonstrates that carbon fibers function beyond passive supports, acting as conductive and structurally adaptive frameworks that govern metal dispersion, interfacial bonding, and electronic coupling. The findings highlight carbon fibers as scalable, material-efficient platforms for designing advanced metal–carbon hybrid systems, in which performance is governed by substrate-driven control of interfacial processes. Although the present investigation was performed using a model ammonia-containing alkaline electrolyte, the obtained results provide important mechanistic insights into catalyst design for future alkaline wastewater electrochemical systems. Validation under real industrial wastewater conditions has been performed separately as part of an ongoing scale-up investigation and will be reported independently.

## Supplementary Information

Below is the link to the electronic supplementary material.

Supplementary Material 1

## Data Availability

The data supporting the findings of this study are available within the article. Additional data are available from the corresponding author upon reasonable request.
