# Supplementary material for: Carbon fibers as active substrates for engineering Ni/Au interfaces toward enhanced hydrogen evolution in ammonia-containing alkaline electrolytes
Source: Sci Rep. 2026 Sep 5;16:27820. doi: 10.1038/s41598-026-70216-y (PMC13546290; doi:10.1038/s41598-026-70216-y)
Supplement: Supplementary file 1 [file 41598_2026_70216_MOESM1_ESM.pdf]

# Supporting Information

# Carbon Fibers as Active Substrates for Controlling Metal Dispersion and Interfacial Charge Transfer in Ammonia-Containing Alkaline Electrochemical Systems

**Annu Pandey,[a] Anton Bjurström,[a,b,g] Dhirendra Sahoo[a], Yuanyuan Li,[a] Valerio Pugliese,[d] Jyoti Shakya,[h] Kåre Tjus,[c] Anna Karlsson,[f] Sudip Chakraborty,[d,e] Jörgen Ejlertsson,[f] Anders Björk[c] and Richard T Olsson [a*]**

[a] KTH – Royal Institute of Technology, Fibre and Polymer Technology, School of Chemical Science and Engineering, Teknikringen 56-58, 100 44 Stockholm, Sweden.
[b] Wallenberg Initiative Materials Science for Sustainability, Department of Fibre and Polymer Technology, KTH Royal Institute of Technology, Stockholm, SE-106 91 Stockholm, Sweden.
[c] IVL Swedish Environmental Research Institute, Stockholm, Sweden.Email:
[d] Department of Computer Engineering, Modeling, Electronics and Systems (D.I.M.E.S.), University of Calabria, Via P. Bucci, Cubo-42A, 87036 Rende (CS), Italy
[e] NANOTECHNOLOGY INSTITUTE (CNR-NANOTEC), Via.P. Bucci, Cube 31/c, 87036 – Rende (CS), Italy.
[f] St1 Biokraft, Kungsbron 1, 111 22 Stockholm, Sweden.
[g] NKT HV Cables, Technology Consulting, Västerås, SE-72178 Sweden.
[h] School of Interdisciplinary Studies & Research, D Y Patil International University, Akurdi, Pune 411044, India.
[i] **Email Corresponding Author Richard T. Olsson: rols@kth.se**

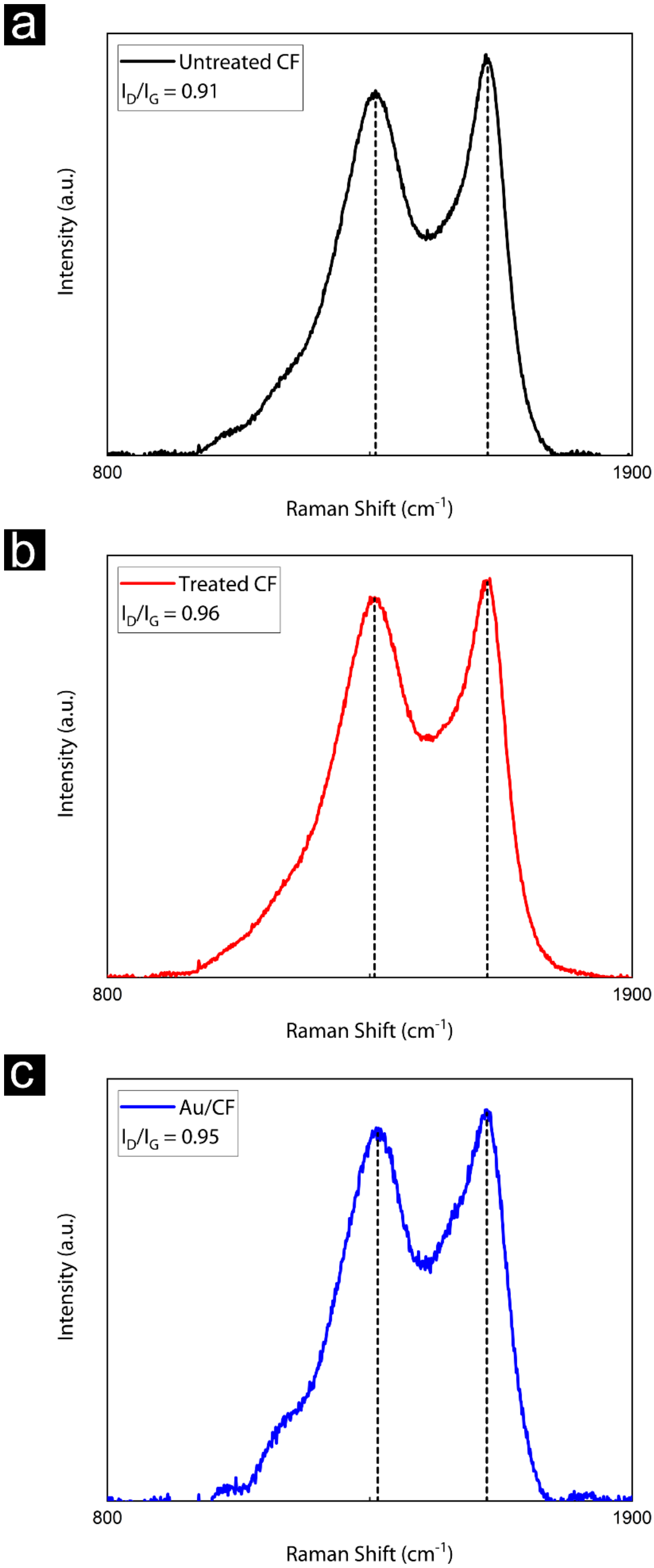


Figure S1. Baseline-corrected Raman spectra used for ID/IG analysis of (a) untreated carbon fiber (UTCF), (b) thermally desized carbon fiber (HVCF), and (c) Ni/Au-coated carbon fiber (Ni/Au/CF)
